# The risk factors of bone metastases in patients with lung cancer

**DOI:** 10.1038/s41598-017-09650-y

**Published:** 2017-08-21

**Authors:** Yang Zhou, Qing-Fu Yu, Ai-Fen Peng, Wei-Lai Tong, Jia-Ming Liu, Zhi-Li Liu

**Affiliations:** 1 Department of Orthopaedic Surgery, the First Affiliated Hospital of Nanchang University, 330006 Nanchang, P.R. China; 2 School of Humanities, Jiangxi University of Traditional Chinese Medicine, 330006 Nanchang, P.R. China; 3 Department of Orthopaedic Surgery, People’s Hospital of Nanhai District of Foshan City, 52800 Foshan, P.R. China

## Abstract

The risk factors of bone metastasis in patients with lung cancer are still unclear. Here, a retrospective study including a series of consecutive patients who were diagnosed with lung cancer between January 2005 and November 2016 was carried out. A total of 2021 patients with lung cancer were included in this study and 23.9% of them were found to be bone metastases. For patients with bone metastases, adenocarcinoma (62.1%) was the most common pathological subtype, and rib (62.3%) was the most frequent distant metastatic site, followed by thoracic (53.8%) and lumbar spine (40.4%). The histopathologic type, CA-125 level and the concentration of alkaline phosphatase (ALP) were identified as the independent risk factors for bone metastases in lung cancer (*P* = 0.002, *P* = 0.001 and *P* < 0.001). The sensitivities and specificities of diagnosing bone metastasis by CA-125 were 32.1% and 80.8%, and by ALP were 41.3% and 77.1%, respectively. Thus, the incidence of bone metastases in lung cancer patients was relative high, and physicians should pay attention to the histopathologic type, the serum CA-125 and ALP concentrations when patients were firstly diagnosed with lung cancer for early detecting bone metastases.

## Introduction

Lung cancer has a high morbidity and mortality worldwide^1–3^. Although the survival rate is increasing after neoadjuvant and surgical treatment nowadays, patients with metastatic disease still have a poor prognosis, especially in those with bone metastases^4–6^. Bone is the most frequent site of distant metastases in lung cancer. The presence of bone metastases is a negative prognostic factor for patients with lung cancer. Santini Daniele *et al*.^7^ found that patients affected by non-small cell lung cancer with bone metastases represented a heterogeneous population in terms of risk of skeletal events. The median survival time of patients with bone metastases is usually less than 1 year from diagnosis of bone metastasis to death^8, 9^. Also, the bone diseases are often complicated by skeletal-related events, including radiotherapy, pathological fractures, spinal cord compression and hypercalcemia. Moreover, these skeletal-related events will decrease patients’ quality of life by causing pain, with decline in physical, functional and emotional well being. Moreover, survival improvement in patients suffered from lung cancer has resulted in a further raise in the incidence of bone metastases and in all likelihood a change in the natural history of this condition.

It is clear that early diagnosis and prevention of bone metastases becomes a relevant clinical intervention to increase the survival rate of patients. Although there are different studies showing that bone metastases definitively influence the overall survival in lung cancer, the risk factors dedicated to bone metastases in lung cancer patients remain unclear. And the data about lung cancer available in literature are less of clear explanation and only base on relatively small cohorts of patients. Therefore, it is necessary to determine the risk factors correlated with lung cancer metastasis to bone for preventing and early treating bone metastasis.

In this retrospective study, we evaluated the correlation between several clinical-pathological factors and bone metastases at the time of diagnosis, and determined the risk factors for bone metastases in lung cancer based on a large population investigation.

## Materials and Methods

### Ethics statement

The ethical committee of the First Affiliated Hospital of Nanchang University has approved this study. Informed consent was obtained from all the patients included in this study. The methods were carried out according to the approved guidelines.

### Study design

A series of consecutive patients who were diagnosed as lung cancer at the First Affiliated Hospital of Nanchang University between January 2005 and November 2016 were included in this study. All the diagnoses were confirmed by the histopathologic examination of the samples harvested from biopsy or surgery. The patients included in this study were firstly diagnosed as primary lung cancer, and admitted to the hospital without radiotherapy or surgery. Patients who were identified as secondary lung cancer were excluded from this study.

The bone metastasis was identified based on one of the following criteria: the identification of bone metastasis was made by radionuclide bone scan; bone metastasis was found by other imaging assessment (e.g. standard x-rays, computed tomography scans and magnetic resonance imaging of the skeleton).

All the medical records of these patients were carefully reviewed, and the demographic and characteristic data of patients at the first diagnosis (before patients undergoing any treatment, such as radiotherapy, chemotherapy and surgery) were collected. The serum concentrations of biomarkers, such as CA-125, CA-199 and alkaline phosphatase (ALP), were recorded. According to the criteria developed by the Department of Nuclear Medicine of the hospital, 27 U/ml was considered to be the upper normal limit for CA-199 and 35 U/ml for CA-125. For ALP, the concentrations of it were divided into three groups according to Wymenga LF *et al*.^10^ classification (<90 U/L, 90–140 U/L and >140 U/L). The correlation between clinical-pathological parameters, biomarkers and bone metastases in these patients with lung cancer at the first diagnosis were analyzed.

### Statistical analysis

The continuous data in this study, such as age, concentrations of CA-125 and CA-199 were firstly divided into subgroups, and then transformed into dichotomous data for analysis. All the clinical parameters were evaluated as possible risk factors related to bone metastases by *Chi* square test. Binary logistic regression analysis was then used to identify the risk factors for bone metastases. The accuracy of diagnosis by the risk factors was calculated by the area under the receiver operating characteristic (ROC) curves. Statistical analysis was conducted by SPSS software (version 19.00; SPSS, Chicago, IL). *P* value less than 0.05 was considered to be statistically significant.

## Results

### Demographic characteristics

A total of 2021 patients were included in this study. The clinical-pathological parameters of patients are displayed in Table 1. Majority of the included patients were male (72.7%). The most common pathological subtype was adenocarcinoma (43.8%), followed by squamous cell carcinoma (34.7%) and small cell lung cancer (12.5%). A total of 483 patients (23.9%) were identified with bone metastases at the time of diagnosis. The ages of patients with and without bone metastases at diagnosis were 59.7 years (range, 28–88 years) and 60.9 years (range, 6 to 96 years), respectively.

**Table 1:** Characteristics of patients with lung cancer included in this study.

Patient characteristics	Number of patients (%) (n = 2021)
Gender	
Male	1469 (72.7)
Female	552 (27.3)
Age at diagnosis(years)	
Mean	60.58 ± 10.74
Range	6–96
Histopathology, n (%)	
Adenocarcinoma	885 (43.8)
Squamous cell carcinoma	701 (34.7)
Small Cell Lung Cancer (SCLC)	253 (12.5)
Large cell carcinoma	70 (3.5)
Poorly differentiated	49 (3.1)
Adenosquamous carcinoma	12 (0.6)
Others	83 (4.1)

In patients with bone metastases, non-small cell lung cancer represented the most common histopathologic subtype (410 cases, 84.9%), which included adenocarcinoma (62.1%), squamous cell carcinoma (21.3%), large cell carcinoma (0.8%) and adenosquamous carcinoma (0.6%) (Table 2).

**Table 2:** The differences between patients with and without bone metastases.

Clinical factors	BM* (n)	NBM** (n)	*P*
Gender			0.344
Man	343	1126	
Female	140	412	
Age (years)			0.013
<60	244	678	
≥60	239	860	
Histopathology			<0.001
Adenocarcinoma	300	585	
Squamous cell carcinoma	103	598	
Small Cell Lung Cancer (SCLC)	40	213	
Poorly differentiated	15	55	
Large cell carcinoma	4	13	
Adenosquamous carcinoma	3	9	
Others	18	65	
CA-125 (U/ml)			<0.001
<35	217	845	
≥35	266	693	
CA-199 (U/ml)			<0.001
<27	242	945	
≥27	130	261	
ALP (U/L)			<0.001
<90	231	992	
90–140	151	398	
>140	95	119	

^*^BM: bone metastases group; **NBM: non bone metastases group.

### Distribution of bone metastases sites

The distribution and number of metastatic sites in patients with bone metastases are demonstrated in Table 3. The most common site of bone metastases was rib (62.3%), followed by thoracic (53.8%) and lumbar spine (40.4%). The least affected site was ulna and radius (0.8%). Moreover, 41.8% of the patients were affected by one site and 38.5% had more than three metastatic sites. This indicates that lung cancer patients have a high incidence risk to get multiple bone metastases.

**Table 3:** The distribution and number of metastatic sites in patients with bone metastases.

Patient characteristics	Number of patients (%) (n = 483)
Site of bone metastases	
Spine	
Cervical	52 (10.8)
Thoracic	260 (53.8)
Lumbar	195 (40.4)
Rib	301 (62.3)
Pelvis	148 (30.6)
Femur	71 (14.7)
Sternum	53 (11.0)
Clavicle	40 (8.3)
Skull	32 (6.6)
Scapula	31 (6.4)
Humerus	24 (5.0)
Tibia and fibular	9 (1.9)
Ulna and radius	4 (0.8)
Number of metastatic sites (n)	
One site	202 (41.8)
Two sites	95 (19.7)
Three and more sites	186 (38.5)

### The correlation between clinical factors and the number of metastases sites

Linear regression analysis was used to determine the correlation between different clinical factors and the number of metastases sites in lung cancer patients. The results showed that both of the concentrations of CA-125 and ALP were correlated with the number of bone metastases sites, respectively (R = 0.113, *P* = 0.012 and R = 0.157, *P* < 0.001).

### Risk factors for bone metastases in patients with lung cancer

In order to identify the risk factors for bone metastases in patients with lung cancer, the clinical factors were compared between patients with and without bone metastases (Table 2). Significant differences were found for patients’ age, histopathologic type and the concentrations of CA-125, CA-199 and ALP between the two groups. Binary logistic regression analysis additionally indicated that histopathologic type (OR = 1.160, *P* = 0.002), the concentration of CA-125 and ALP (OR = 0.999, *P* = 0.001 and OR = 0.996, *P* < 0.001) were the independent risk factors for bone metastases in lung cancer (Table 4).

**Table 4:** The risk factors of bone metastases in patients with lung cancer.

Factors	B	OR	OR (95% CI)	*P*
Histopathology	0.148	1.160	1.055–1.274	0.002
CA-125	−0.001	0.999	0.999–1.000	0.001
ALP	−0.004	0.996	0.995–0.998	<0.001

CI: confidence interval; OR: odds ratio; B: coefficient of regression; ALP: alkaline phosphate.

### The sensitivities and specificities of risk factors for diagnosing bone metastases

The area under ROC curves (AUC) was analyzed to determine the accuracy of CA-125 and ALP levels on diagnosing bone metastases in patient with lung cancer. The AUC values were 0.588 for CA-125 and 0.610 for ALP, respectively. The sensitivities and specificities for the diagnosis of bone metastases by CA-125 were 32.1% and 80.8%, and by ALP were 41.3% and 77.1%.

## Discussion

Distant metastasis is one of the main features of malignant tumor. In consistent with other malignant diseases, lung cancer is also prone to metastasis, and bone is the common site of distant metastasis for it. More and more studies show that lung cancer is the third most frequent form of cancer causing malignant lesions of bone metastases^11, 12^. It is reported that the prevalence of bone metastasis second from lung cancer at the time of diagnosis is 15–40%^11, 13, 14^. In the present study, the incidence of bone metastases at initial diagnosis was 23.9% in patients with lung cancer, and adenocarcinoma (62.1%) was found to be the most common histopathologic subtype, which was similar to the results of previous studies^11^.

For the distribution of bone metastases from lung cancer, few studies reported the exact outcomes of it. Based on a large population analysis in our study, the most frequent site of bone metastases was rib (62.3%), followed by thoracic (53.8%) and lumbar spine (40.4%). These results are consistent with Quint *et al*.'s study^15^. The reasons for the high incidence of rib and thoracic spine metastases in lung cancer patients are the existence of venous traffic branches among lung, intercostals and vertebral vein, and the short distance among these organs. Thus, the lung cancer cells can easily spread to ribs and thoracic spine. Moreover, we found the incidence of multiple sites of bone metastases was relative high in patients, which was just second to single site metastasis. And the concentrations of CA-125 and ALP were detected to be associated with the number of bone metastases sites. It means that the higher levels of CA-125 and ALP are, the more possibility of multiple bone metastases may occur.

Several studies have reported the risk factors for bone metastases second from lung cancer. Oliveira *et al*.^11^ found that the histological subtype of adenocarcinoma was significantly associated with higher risk of developing bone metastases in patients with lung cancer. Conen *et al*.^16^ showed that elevated LDH and age ≥75 years were independent risk factors for bone metastases in small cell lung cancer patients. In accordance with previous studies, we also found histopathologic type was one of the risk factors for bone metastases in lung cancer patients. However, different from previous studies, we additionally identified the concentrations of CA-125 and ALP as the independent risk factors of bone metastases.

The tumor marker CA-l25, which is specific as a marker of malignant disease, has been applied for diagnosis and evaluation recurrence of ovarian cancer more than 30 years. Perioperative serum CA-125 level is a prognostic factor for survival in patients with ovarian cancer^17^. Studies carried out by Chung *et al*.^18^ and Son *et al*.^19^ revealed that elevated CA-125 level was associated with advanced stage disease and lymph node metastases in endometrial cancer. Han *et al*.^20^ and Chao *et al*.^21^ proposed different cut-off values for predicting lymph node metastases in patients with endometrial cancer. CA-125 is not only produced by epithelial tumors, but also by mesothelial cells. Thus, the level of CA-125 would be higher in pleural fluid than that in serum^22^. Choi *et al*.^23^ found that pleural CA-125 levels were significantly higher in malignant pleural effusions than those in benign pleural effusions. Bielsa *et al*.^24^ indicated that high level of CA-125 in pleural fluid was an independent predictor of poor outcome in squamous malignant effusions. However, few studies investigated the correlation between serum CA-125 level and bone metastases in lung cancer currently. In the present study, we retrospectively evaluated the serum concentration of CA-125 in 2021 lung cancer patients with and without bone metastases. The results showed that the serum CA-125 level was significant higher in patients with bone metastases compared to those without bone metastases. And it was an independent risk factor for bone metastases in lung cancer, with a sensitivity of 32.1% and specificity of 80.8%.

ALP is presented in many human tissues, such as bone, kidney and liver. It partly reflects the activity of osteoblast and can be used as a marker for patients with larger volume or aggressive bony metastatic disease. Studies indicated that the serum ALP level was associated with bone metastasis in prostate cancer, and high level of it was related to a poor clinical outcome^25, 26^. Alataş *et al*.^27^ reported that the level of ALP was significantly higher in lung cancer patients with bone metastases than those without bone metastases, and the sensitivity and specificity of it for diagnosing bone metastases were 89% and 44%. Kang *et al*.^6^ found that high level of ALP was an independent poor prognostic factor for small cell lung cancer with bone metastases. In line with Kang *et al*. study, we also detected that ALP was a risk factor for bone metastases in lung cancer patients. The sensitivity and specificity for diagnosing bone metastases by it were 41.3% and 77.1%. Thus, our results indicate that serum ALP level may be useful to predict bone metastases in patients with lung cancer.

Although we successfully identified the independent risk factors for bone metastases in lung cancer, there are still some limitations in the present study. First, it was a retrospective study and some clinical parameters were not included in the analysis because of insufficient data, which may affect the outcomes of this study. Second, we just reviewed the data of patients at the time of diagnosis, and some data such as time to develop bone metastases, survival rate of patients and the duration of follow-up were not reported in this study. Third, only patients from a single institution were involved in this study. And a prospective, multi-center study is necessary to verify the results of our study.

In conclusion, based on a large population investigation, the incidence of bone metastases in lung cancer was 23.9%, and the most frequent bone metastases sites were rib and thoracic spine in our study. The most common histopathologic subtype was adenocarcinoma in patients with bone metastases. Histopathologic type and the serum concentrations of CA-125 and ALP were identified as the independent risk factors of bone metastases in lung cancer. However, some limitations are existed in this study and a prospective, multi-center study is necessary to verify the results.
